# Lessons in Physical Diagnosis

**Published:** 1872-12

**Authors:** 


*Lessons in Physical Diagnosis.* By ALFRED L. LOOMIS, M. D. New York: Wm. Wood & Co., 1872; Buffalo: H. H. Otis.

The work of Dr. Loomis on Physical Diagnosis has been for some time a book of reference to student and practitioner. Clear where the student might be in danger of misapprehension, ample in illustration, and concise in direction, it has always been a favorite with all. The new edition, therefore, will be welcomed with no small amount of pleasure by the army of graduates who are shortly to go out to practice auscultation, percussion, and the other means of physical diagnosis, upon suffering mankind. To the new edition there have been added five lessons,—three upon examination of the urine as applied to diagnosis, and two on the mechanical aids to diagnosis. The original text has also been revised and enlarged, and the author presents to us in the third edition of his work a guide in diagnosis which seems in every respect complete. The present edition as enlarged comprises over two hundred and thirty pages, and it is issued in a form at once elegant and serviceable. We know of no work of more value to student or young practitioner.

